# Violence Against Women in Spanish Prisons: A Socio-Educational Analysis from a Sustainable Development Perspective

**DOI:** 10.3390/bs16050731

**Published:** 2026-05-09

**Authors:** Fanny T. Añaños, Álvaro Moraleda-Ruano, Diego Galán-Casado, Alina D. Corpodean

**Affiliations:** 1 Faculty of Educational Sciences, University of Granada, 18011 Granada, Spain; fanntab@ugr.es; 2 Faculty of Education, University Camilo José Cela, 28692 Madrid, Spain; amoraleda@ucjc.edu; 3 Faculty of Education, National University of Distance Education (UNED), 28040 Madrid, Spain; diegog@edu.uned.es; 4 Doctoral Programme in Education, University of Granada, 18011 Granada, Spain

**Keywords:** gender-based violence, abuse, prison, gender equality, prison education, socio-educational intervention, risk factors, protective factors

## Abstract

This study analyses gender-based violence across the life courses of women serving prison sentences in Spain, adopting a socio-educational and gender-based approach grounded in social sustainability. It is a quantitative, descriptive, and interpretative study of a sample of 310 women in semi-liberty from 31 prisons, using semi-structured questionnaires. The study identifies the presence of different forms of intimate partner abuse and examines their relationship to several variables. The results show that 47.44% of the women have experienced some form of abuse, a higher percentage than the national average. Having a stable partner, having been employed prior to imprisonment, and participating in educational activities, daily life skills programmes, and socio-educational or gender-focused interventions are associated with a lower prevalence of abuse. Substance addictions and having received disciplinary sanctions, in contrast, are associated with greater exposure to violence, while motherhood and recidivism show no significant association. These findings reveal the presence of structural and sociocultural violence and highlight the need for coherent prison policies, development of quality education, gender equality, and actions to improve coexistence and social justice by promoting social reintegration and advancing social sustainability through the prevention of violence and social exclusion.

## 1. Introduction

Violence is a complex, heterogeneous phenomenon, in both its manifestations and how it is perceived. Violence can also have tremendous consequences socially and for public health (World Health Organization, 2002). Because violence is challenging to define, it has always been treated from varying perspectives. Among the most prominent is that of the sociologist Johan Galtung, a pioneer in the sociology of peace and conflict, who distinguishes between direct, structural, or indirect, and cultural violence (Galtung, 1969, 1998). Belsky (1993), in contrast, refers to child abuse as violence based on Bronfenbrenner’s ecological model (Bronfenbrenner, 1979). This model is associated with the risk factors present in various systems and stresses a violent or negative relationship with parents as a risk factor in childhood development (Bronfenbrenner & Morris, 2006; Sirparanta et al., 2024). Žižek (2017) differentiates between subjective and objective violence, terms similar to Galtung’s direct and indirect violence, respectively. Finally, some contemporary authors find that the perception of the word “violence” has evolved, broadening the concept beyond physical harm to include structural or symbolic violence linked to social justice and inequalities (Van Breen et al., 2025), as well as contexts of socioeconomic vulnerability and precarity (Khofi et al., 2025) in which multiple axes of inequality converge (Crenshaw, 1989). From this perspective, gender-based violence can be understood as an expression of structural or symbolic inequality derived from the reproduction of the asymmetrical power relationships between men and women and widely recognized as one of the most persistent and serious manifestations of abuse in our societies.

Abuse of women, better known in the Spanish context as gender-based violence, involves an imbalance in gender roles. The man exerts control over the woman through means ranging from multiple coercive mechanisms to physical and/or other types of violence (Flood, 2019; Schraiber et al., 2010; World Health Organization, 2002). Such violent exercise of power prioritizes masculine sexual interests over feminine to justify the violence imposed (Balint, 2024). In the current Spanish context, gender-based violence is regulated by Organic Law 1/2004 and defined as violence that occurs in an intimate relationship or outside it with an ex-partner, and that is exerted by the man in a heterosexual relationship.

Gender-based violence has significant consequences for the victim’s physical and mental health. Anxiety, depression, sexually transmitted diseases/infections, suicidal ideation, and post-traumatic stress are only some of the most common symptoms (Devries et al., 2013; Dunkle et al., 2004; Puente-Martínez et al., 2015; Smith et al., 2020). Gender-based violence is also a social and public health problem, as it impacts not only women but their entire immediate environment (children, family members, work, etc.) (Macias-Bowen & Macias-Bowen, 2022; Schraiber et al., 2010) and society in general.

A wide range of risk factors associated with gender-based violence have been identified, including emotional dependence, substance use, fear, motherhood, deteriorating mental health, lack of family and community support, economic difficulties, masculine cultures and cultures of honour, not having a job or housing, and having a low level of education or training, among others. All these factors influence and continue to reproduce situations of vulnerability for women (F. T. Añaños et al., 2022; Puente-Martínez et al., 2015). Such is the case in our study, which focuses on women serving prison sentences (Jones et al., 2021).

### 1.1. Gender-Based Violence Among Incarcerated Women

The prison context shows life experiences plagued with violence, even before people go to prison. Around 88.4% of the women in prison have been victims of some type of violence: 68% have experienced sexual violence, 41% of these repeatedly; 59% have experienced violence in their family environment, and 25% during childhood. Further, 74% have suffered physical violence, 80.4% of whom were abused or lived in situations with domestic violence—a figure considerably higher than the 12.4% estimated for the general population (Secretaría General de Instituciones Penitenciarias [SGIP], 2016). Adverse experiences of violence during childhood also influence women in prison, as 57% of women and 46% of men state that they were exposed to violence and trauma prior to going to prison (Clark & Duwe, 2025). The SGIP’s most recent study (2023) of the profile of women victims of gender-based violence in prison observes that 69.7% of women in prison suffered psychological violence, 61.4% physical violence, and 46.4% sexual violence.

As most of these cases were perpetrated by the intimate partner or ex-partner, conditions of social, cultural, educational, and economic disadvantage and social conditions of the women’s natural environment before going to prison determine and increase the likelihood of such circumstances and multiple forms of exclusion (F. T. Añaños, 2012b; Richie, 2001; Secretaría General de Instituciones Penitenciarias [SGIP], 2023). In the same vein, Quiroga-Carrillo and Lorenzo-Moledo (2025) stress gender-based violence and child abuse as accumulated victimization that conditions the lives of women in prison, while also being tied to structural factors, such as a low education level or drug use. This finding agrees with the literature on intergenerational transmission of violence or cycles of violence, which shows that exposure to violent experiences at an early age can shape subsequent paths of victimization and exclusion (Widom & Wilson, 2015). A partner can also be a protective factor, however, preventing crime or criminal recidivism. Partners, as well as other factors such as family support or job stability (Galán-Casado, 2015; Galán-Casado et al., 2021; Gallizo, 2007; Martín Artiles et al., 2009), provide emotional support that improves personal and social well-being (F. T. Añaños et al., 2022; Cobbina et al., 2014; García-Vita & Rivera-López, 2022; Giordano et al., 2011; O’Brien, 2001; O’Connor et al., 2024).

This distinction is especially relevant to our study, which addresses gender-based violence as a broader cumulative phenomenon shaped by structural, sociocultural, and life-course factors. In contrast, the empirical analysis focuses specifically on abuse experienced in the context of a relationship with an intimate partner or former partner.

### 1.2. Gender-Based Violence and Education

F. T. Añaños (2013, 2022) and F. T. Añaños et al. (2020) argue that women in prison suffer what is known as structural violence (Galtung, 1969, 1998), forms of violence exerted by social and institutional structures that prevent certain groups from satisfying their specific needs. From this sociological perspective, the prison system’s rehabilitation or motherhood support programmes are limited. So are training/employment offerings (among others), perpetuating gender inequalities and limiting access to sufficient resources for the women’s rehabilitation and integration (Burgos-Jiménez, 2022; Burgos-Jiménez et al., 2023; Gallizo, 2007; Herrera & Expósito, 2010; Viedma & Del Val, 2019). These conditions produce relationships inferior to those of the men’s prison population due to the presence of fewer women in the prison system, less availability of resources and programmes for them, and little adaptation of existing resources to women’s needs. These issues contribute to distorting, hierarchizing, intensifying, and institutionalizing women’s situation of disadvantage (García-Vita & Añaños, 2021).

In this framework, gender-based violence emerges as a complex multicausal phenomenon affected by various personal and structural dynamics that become factors sustaining unequal power relationships (F. T. Añaños et al., 2022; García-Vita & Rivera-López, 2022).

To address the need to eradicate violence—particularly gender-based violence in its multiple forms—and ensure social well-being, the 2030 Agenda for Sustainable Development includes Sustainable Development Goal 5 (SDG 5), on achieving gender equality and empowering all women and girls (F. T. Añaños, 2022; F. T. Añaños et al., 2022; United Nations, 2015). This goal promotes the protection of fundamental human rights, recognizes the value of care and domestic work traditionally performed by women due to assigned gender roles, encourages women’s full participation and leadership in the social and political spheres, ensures economic rights and universal access to sexual and reproductive health, and guides the adaptation of policies and legislation towards the elimination of all forms of gender-based discrimination.

Because prisons are spaces shaped by processes of social exclusion and multiple forms of violence (F. T. Añaños, 2012b; Secretaría General de Instituciones Penitenciarias [SGIP], 2023), this goal is articulated from a gender perspective in the prison context in close connection with Sustainable Development Goal 4 (Quality Education) (United Nations, 2015). Recognition of the universal right to education in prison thus becomes a central axis for re-education, representing one of the main challenges in the Spanish prison system, to promote effective social reintegration and prevent recidivism (K. Añaños et al., 2019; LOGP, 1979).

In this regard, it is vitally important to identify and implement prosocial strategies that reduce violence and situations of exclusion, promoting the development of human values and contributing to the construction of a more egalitarian society (F. T. Añaños, 2012b, 2022; F. T. Añaños et al., 2022). Education and Social Pedagogy are crucial fields of action and research for the design and implementation of socio-educational interventions to foster processes of social transformation in complex contexts such as prisons (F. T. Añaños, 2012a).

In this framework, women’s lower education level emerges as a significant risk factor for gender-based violence, while a higher education level is a protective factor. Early school leaving is also very common among women in prison (66.6%). Only 29.2% have completed secondary education, vocational training, or higher education. Traditional gender roles associated with domestic care and greater dependence on a partner also contribute to this situation due to the lack of paid employment (F. T. Añaños et al., 2020).

Prison is thus a key place in which to foster personal autonomy and implement socio-educational actions to meet the needs of life-courses marked by recurrent episodes of violence (Quiroga-Carrillo & Lorenzo-Moledo, 2025). Such actions have led to improvement in the educational paths of women deprived of liberty. These paths are associated with greater participation in formal education (57%), which in turn contributes to emotional well-being and facilitates future entry into the labour market following release (F. T. Añaños et al., 2020).

As to the implementation of socio-educational and gender-specific programmes, 46% of women deprived of liberty in a Spanish sample participated in these interventions. SerMujer.es, which focuses on gender-based violence and women’s empowerment, has a participation rate of 52.6%. The programme was implemented in 22 prisons in 2024, with an average annual participation of 172 individuals. Other similar programmes include the Equality Promotion Programme (46.1%) and other programmes with a participation rate of 4.6% (F. T. Añaños et al., 2022; Secretaría General de Instituciones Penitenciarias [SGIP], 2024).

As to men deprived of liberty who participated in violence-related programmes in 2024 (Secretaría General de Instituciones Penitenciarias [SGIP], 2025), in contrast, 1299 participated in the Intervention Programme for Gender Violence Offenders (PRIA) across 60 centres, 572 in the Sexual Aggression Control Programme (PCAS) in 48 facilities, 15 in programmes related to child pornography offences in four centres, and 220—along with 24 women—in the Violent Behaviour Control Programme (PICOVI) across 31 prisons.

Women in prison face significant difficulties accessing treatment or rehabilitation programmes that address their specific needs, due to a lack of resources or approaches that fail to incorporate a gender perspective (Galán-Casado et al., 2024; Moles-López & Añaños, 2021; Secretaría General de Instituciones Penitenciarias [SGIP], 2023; Turbi & Llopis-Llacer, 2017).

Moreover, prison often becomes an environment in which violence not only persists but also increases (Secretaría General de Instituciones Penitenciarias [SGIP], 2023). The same occurs if we compare gender-based violence among incarcerated women to the general average (Fernández-Iglesias, 2017). In the general Spanish population, official data indicate that registered cases of gender-based violence account for approximately 0.16% (INE, 2025).

This study thus aims to explore the relation of situations of gender-based violence, expressed as abuse of women, in Spanish prisons to different variables and risk factors (partner influence, motherhood, substance use, behaviour in prison, and penitentiary recidivism, among others), with special attention to participation in educational and socio-educational programmes. From a social sustainability perspective, the study seeks to identify the factors influencing this problem in order to address them through treatment and socio-educational action in prison. Such action would foster processes of social reintegration and contribute to real gender equality for women in this context. We understand gender-based violence as a multicausal phenomenon produced by the interaction of structural, sociocultural, and personal factors that intensify in the prison context and can be addressed in part through socio-educational intervention.

## 2. Materials and Methods

This study follows a quantitative design that is descriptive and interpretive. It analyses gender-based violence against women in the Spanish prison context. The following describes the goals, methods, design, sample, instruments, procedure, and data analysis used.

### 2.1. Goal

This study analyses gender-based violence, specifically the presence of different forms of intimate partner abuse in women serving sentences in the Spanish prison system. We seek to understand how factors such as a stable partner, motherhood, work experience, and participation in educational activities influence the perception of having been a victim of abuse. We adopt a socio-educational perspective oriented to social sustainability and reintegration. It is important to stress that many of these women have experienced abuse before going to prison, and this experience can affect their behaviour both inside and outside the prison environment. We hope this analysis will contribute to the development of more effective interventions and public policies to improve the well-being and mental health of women in prison. Such interventions would encourage these women’s social reintegration and reduce the risk of future episodes of violence.

### 2.2. Design

The study adopts an ex post facto (non-experimental) design suitable for analyzing relationships between variables when the independent variables cannot be manipulated because they have already occurred (Campbell & Stanley, 1963; Kerlinger, 1987; Mateo, 1997). Although this design does not allow us to establish causality, we interpret the associations identified from a socio-educational and gender perspective that views violence as a complex phenomenon linked to individual, social, cultural, economic, and structural factors, among others. The quantitative and interpretive study was performed within the framework of the Spanish national R&D&I project “Reintegration and support processes for women on temporary release” (REINAC) (Ref. EDU2016-79322-R).

### 2.3. Population and Sample

Sampling was conducted in two stages. The first chose representative regions in Spain with the largest number of women serving sentences in the open regime. The second performed simple random sampling of the women who wished to participate in the study, with the requirement that the women had been in prison for at least six months. The fieldwork was conducted from June 2018 to March 2019 in 30 prisons located in 13 of Spain’s 17 autonomous communities. The target population was women serving open regime sentences in Spain (3rd and special 2nd degree). The two-stage sampling yielded a valid sample of 310 women, representing 30.1% of the total population of women in Spanish prisons. Participants’ average age was 42.19 years (SD = 10.678). The average sentence length was 66.05 months (SD = 52.003). Table 1 summarizes the sociodemographic data for the sample.

The sample showed heterogeneity in variables such as national origin, marital status, and context in which the sentence was served, reflecting diversity in the life-courses of the women in semi-liberty.

### 2.4. Instruments

We used a mixed questionnaire designed ad hoc because this instrument combines closed questions for quantitative analysis with open ones for qualitative analysis. The survey was composed of 115 questions divided into 6 sections. The variables were selected from the questionnaire that best agreed with our study goals and focused on the experience of abuse in relation to various factors. We measured the variable “abuse” through participants’ self-perception and identification of violent acts in the context of an intimate partner or ex-partner. This method enabled us to define different types of violence (physical, psychological, and sexual).

We included the factors marital status, motherhood, and substance dependency (e.g., tobacco or alcohol consumption, participation in a Methadone Maintenance Programme [MMP]). We also analyzed having a job in the year prior to going to prison and involvement in educational or socio-educational programmes in prison. For this last case, we considered primary and secondary education received, learning daily living skills, and participation in socio-educational or gender-based programmes. We also examined recidivism and disciplinary sanctions during the women’s time in prison. The instruments were validated through a pilot test of 19 questionnaires and 12 interviews, as well as by a group of 15 experts (academics and professionals).

### 2.5. Procedure

The study was approved by the Ethics Commission of the SGIP and the Department of Justice of the Government of Catalonia. It followed the standards of the University of Granada’s Code for Best Research Practices. We first contacted the prisons to coordinate the administration of the survey. Once the women were gathered in a suitable place, we explained the characteristics and implications of the study. Those who chose to participate gave written formal voluntary consent before completing the questionnaires. The study thus fulfilled the ethical principles of the Declaration of Helsinki and the current European regulations (General Data Protection Regulation [GDPR], 2016). Depending on their need, the women completed the questionnaire with guidance, by themselves, or both, based on their reading competence and understanding of Spanish.

### 2.6. Data Analysis

The data were analyzed using Statistical Package for Social Sciences (SPSS) 24 software, with quality controls to guarantee reliability and validity of the results. We performed bivariate analyses using Pearson’s Chi-square test (χ^2^) with continuity correction to determine the relationship between the variables analyzed. We calculated the effect size using the Phi (Φ) coefficient with SPSS 26.0.

## 3. Results

The results show various significant associations among the sociodemographic characteristics of the women in the open regime and the multiple dimensions of abuse.

Around 47.44% of the women in the open regime had suffered abuse—whether physical (38.6%), psychological (49.5%), or sexual (19.6%).

First, the relationship between partner stability and prevalence of abuse took a value of χ^2^ = 5.294 (*p* = 0.021), yielding a moderate effect size (Φ = 0.149) according to Rea and Parker (1992). Effect size ranged from very small <0.10 to large >0.80, indicating a statistically significant relationship between having a stable partner and suffering less abuse. Motherhood, in contrast, showed no significant association with the prevalence of abuse (χ^2^ = 0.286, *p* = 0.593, Φ = 0.076), suggesting that being a mother does not clearly impact experiencing abuse in this group of women (see Table 2).

Abuse was perpetrated mainly by ex-partners, and only 25% of the women had reported these acts.

As to employment (38.9% of the women had jobs; 62.7% had worked in the past year), having a job in the year prior to going to prison was significantly associated with lower prevalence of abuse (χ^2^ = 4.078, *p* = 0.043, Φ = 0.147), with a low-to-moderate effect size. This finding could show that employment increases emotional and economic stability, factors that could be related to lower vulnerability to situations of violence (see Table 3).

Substance use (alcohol and drugs)—47.7% of the women consumed these substances—was significantly related to the prevalence of abuse, with a value of χ^2^ = 7.203 (*p* = 0.007, Φ = 0.175), indicating a moderate effect. The women who reported having substance dependencies (except tobacco, nonproblematic consumption of alcohol, and treatment for psychotropic drugs [MMP]) experienced a greater prevalence of abuse. This finding suggests the importance of including substance dependency as a possible factor of vulnerability in the experience of violence (see Table 4).

We also found a significant relationship between the inmates who had received disciplinary sanctions (22.4% of the women) during their sentence and prevalence of abuse (χ^2^ = 6.759, *p* = 0.009, Φ = 0.183). This finding may indicate a relationship between punitive contexts and dynamics associated with abuse and violence. Recidivism (25.59%) was not significantly related to abuse (χ^2^ = 0.842, *p* = 0.359, Φ = 0.054), however. Thus, committing another crime seems not to be directly related to having been a victim of abuse (see Table 5).

### Education Programmes

Participation in education programmes in the prison system was significantly related to lower prevalence of abuse (χ^2^ = 3.885, *p* = 0.049, Φ = 0.131), although the effect was small. Participation in activities that promote daily living skills was also significant (χ^2^ = 8.040, *p* = 0.005, Φ = 0.166), with a moderate effect. These findings indicate that access to education and the development of daily living skills could be associated with a lower prevalence of abuse. Participation in socio-educational or gender-based programmes was also associated with lower prevalence of abuse (χ^2^ = 7.679, *p* = 0.006, Φ = 0.162), with a moderate effect size (see Table 6).

## 4. Discussion

Our analysis shows the important role of gender-based violence in society, in accord with the evolution of the concept of violence to include factors that produce social inequality (Van Breen et al., 2025) and exclusion (F. T. Añaños, 2012b; Secretaría General de Instituciones Penitenciarias [SGIP], 2023). Such factors manifest more sharply in the prison environment. In fact, some recent studies in international contexts show that violence against women is a stronger risk factor in situations of economic precarity and in survival strategies developed in scenarios of vulnerability (Khofi et al., 2025). These studies partially strengthen the interpretation of the results from a structural perspective.

In Spanish prisons, more specifically, we see a 47.44% prevalence of abuse among women sentenced to prison, as opposed to 0.16% in the general Spanish population (INE, 2025). Abuse is thus a huge problem among incarcerated women.

Our study provides empirical evidence for how different risk and protective factors—such as partner, employment, substance use, or participation in programmes—are related to abuse in women in semi-liberty. The evidence shows the importance of socio-educational intervention to prevent abuse and facilitate social reintegration.

As to forms of abuse, women inmates most often experience psychological and physical abuse, followed by sexual abuse. These data contradict various studies, such as Leone and Beeble (2024) and St. Cyr et al. (2021), which find that approximately 90% of women have suffered some type of psychological violence or physical abuse. Our data agree, however, with SGIP study findings (Secretaría General de Instituciones Penitenciarias [SGIP], 2023) indicating that women prisoners who are victims of gender-based violence have most often experienced psychological violence (69.7%), followed by physical (61.4%), and then sexual violence (46.4%). Quiroga-Carrillo and Lorenzo-Moledo (2025) find that a significant number of women in prison were victims of gender-based violence and abuse during childhood. Whether high or low, all these percentages indicate the need to develop interventions to address both couple dynamics and histories of abuse in prison. Only so can we achieve effective reintegration, given the increase in gender-based violence among women inmates (Fernández-Iglesias, 2017). Further, only 25% of the women say that they have reported the violence, a very low figure relative to the number of women who have suffered gender-based violence.

The results for partner stability and abuse show a significant relationship between some socio-personal characteristics of women in the open regime and prevalence of violence (χ^2^ = 5.294; *p* = 0.021). Having a stable partner was associated with lower prevalence of abuse, underscoring the importance of fostering healthy couple dynamics as part of interventions to reduce violence in vulnerable contexts (F. T. Añaños, 2022; F. T. Añaños et al., 2022; Cobbina et al., 2014; García-Vita & Rivera-López, 2022; Giordano et al., 2011; Schraiber et al., 2010). We must also consider, however, that many women who go to prison have already been exposed to abusive relationships and multiple disadvantages and exclusions, as explained in the previous section (F. T. Añaños, 2013; F. T. Añaños et al., 2020; García-Vita & Rivera-López, 2022; Puente-Martínez et al., 2015).

The results for motherhood show no clear association between being a mother and the prevalence of abuse. The significant relationship between having harmed one’s children and experienced abuse (χ^2^ = 4.078, *p* = 0.043, Φ = 0.147) indicates, however, a possible relationship between family dynamics and perpetuating situations of violence (Garbarino, 1999; Giordano et al., 2011; Moreno García & Moreno Cuahtecontzi, 2023; Sirparanta et al., 2024; Walker, 1979) or the intergenerational transmission of violence in people who experienced violence at an early age. Our evidence thus shows the need to promote specific interventions to address the consequences of exposure to traumatic situations (Herrenkohl et al., 2020; Merino Lorente, 2024).

Substance consumption was associated with a higher prevalence of abuse (χ^2^ = 7.203, *p* = 0.00, Φ = 0.175), with 47.7% of the sample involved in substance consumption. These results agree with those obtained by Yang (2024) and Ataiants et al. (2020), who stress that situations of social exclusion produced by consumption emerge in disruptive contexts where violence is very present. They also agree with the SGIP study (Secretaría General de Instituciones Penitenciarias [SGIP], 2023), which shows 76.7% consumption among victims of gender-based violence, as opposed to 34.5% among non-victims. This situation demonstrates the need to include drug consumption treatment programmes, such as treatment for drug dependency, as a support strategy to fight addiction, and to encourage healthy lifestyles after the women obtain their freedom. Women must thus develop competences, attitudes, and resources to prevent abuse and any type of violence (F. T. Añaños & Burgos-Jiménez, 2024; F. T. Añaños et al., 2022; Burgos-Jiménez et al., 2023; Galán-Casado et al., 2024; Gallizo, 2007; Moles-López & Añaños, 2021; Turbi & Llopis-Llacer, 2017).

Another important finding is the significant relationship (χ^2^ = 6.759, *p* = 0.009, Φ = 0.183) between disciplinary sanctions and abuse. Of the participating women, 22.4% had received sanctions during their sentence. This phenomenon could be interpreted theoretically as a manifestation of structural violence (Galtung, 1969), as the prison system responds punitively to behaviour that may derive from life experiences of victimization. This finding validates Galtung’s sociological approach to understanding how institutional structures can perpetuate less visible but equally damaging forms of violence. From an ecological perspective (Belsky, 1993), these women bring with them risk factors that affect both their going to prison and their adaptation to the prison environment, reproducing dynamics of institutional exclusion (Richie, 2001).

In contrast, the results of Clark and Duwe (2025), based on a broad sample of prisoners in the US, show that women who have previously experienced violence have a harder time adjusting institutionally. This difficulty does not always manifest in sanctionable behaviour, as in the case of men, but rather in more internal forms of emotional suffering. This difference may be due to the specific characteristics of the Spanish prison system, which deprives women and men inmates of freedom differently (Galtung, 1969).

Unlike other variables, recidivism shows no significant association with abuse. We do not observe a direct relationship between having been a victim of violence and recidivism in this sample. This finding highlights the complexity of criminal activity, which is influenced by multiple personal, social, and structural factors (Belsky, 1993; Bronfenbrenner, 1979; Bronfenbrenner & Morris, 2006; Moles-López & Añaños, 2021). Although many women inmates have prior experience of violence (Cruells et al., 2005), its relationship to recidivism seems to be mediated by other elements, such as family support, emotional stability, and access to resources after leaving prison (Giordano et al., 2011), among others.

Employment is an important factor, as women who worked before going to prison (38.9% were employed; 62.7% had worked in the previous year) show lower prevalence of abuse (χ^2^ = 4.078, *p* = 0.043, Φ = 0.147). This finding may be related to the economic and emotional independence that employment provides, which may in turn be related to less vulnerability to situations of violence. These results further reinforce the importance of promoting access to work in the programmes on preparation for freedom (F. T. Añaños et al., 2022; Galán-Casado, 2015; Galán-Casado et al., 2021; Gallizo, 2007; Martín Artiles et al., 2009), as work and economic income are fundamental keys to social reintegration (F. T. Añaños, 2022; F. T. Añaños et al., 2022; Esteban et al., 2014). Employment can also be related to the potentially protective element of support and social networks established in the work environment. These networks serve as protective factors (O’Connor et al., 2024), reinforcing the role of employment as a key element of social sustainability and equality in the reintegration processes of women deprived of liberty.

Despite this positive statistic, it is crucial to change other situations involving prejudices and social stigma, as employers show bias against hiring people who have been in prison (Duwe, 2018; Galán-Casado et al., 2024; García-Jarillo et al., 2016; Lopoo & Western, 2005). Such negative factors are especially concerning in the case of women, who are already hindered by a lack of education, training, and socio-job-related competences (F. T. Añaños, 2013; F. T. Añaños et al., 2020; García-Vita & Rivera-López, 2022; García-Vita & Rivera-López, 2022) that can perpetuate circumstances of social disadvantage and forms of exclusion. We must consider that prisons not only act as places of intervention but can also reproduce dynamics of control and inequality that affect the women in different ways, especially when different axes of inequality converge (Crenshaw, 1989).

The results for participation in educational programmes and activities show that engaging in such programmes influences the development or strengthening of daily living skills, which are associated with a lower prevalence of abuse. These findings are consistent with those reported by Galán-Casado et al. (2024) and Molina et al. (2020), who highlight the capacity of education to identify situations of violence, as well as its role as a tool for personal development in prison contexts.

From this perspective, education becomes a central axis of social sustainability. It helps to reduce inequalities, promote equal opportunities and autonomy, strengthen life-courses less exposed to violence, and foster resilience to generate social well-being and reduce violence (F. T. Añaños, 2022; F. T. Añaños et al., 2022; Herrera & Expósito, 2010; Quiroga-Carrillo & Lorenzo-Moledo, 2025; United Nations, 2015; Viedma & Del Val, 2019). Interventions that promote educational training, skills development, prosocial attitudes, and participation in socio-educational programmes are thus associated with improvements in the well-being of women in semi-liberty and with lower incidence of violence (F. T. Añaños, 2012b; F. T. Añaños et al., 2022). These interventions facilitate women’s social reintegration and reduce the risk of recidivism, consistent with the principle of re-education and social reintegration as one of the fundamental aims of the Spanish penitentiary system (F. T. Añaños, 2013; K. Añaños et al., 2019; F. T. Añaños et al., 2020; F. T. Añaños, 2022; F. T. Añaños et al., 2022; LOGP, 1979; Moles-López & Añaños, 2021). However, we must remember that these interventions are performed in a prison context with structural, organizational, and resource limitations that condition their scope and the possibility of fully guaranteeing quality education in the terms established by SDG 4 (F. T. Añaños, 2022; United Nations, 2015).

## 5. Conclusions

Being a woman and having committed a crime is associated with a process of double social stigmatization. This situation can worsen with the addition of specific problems, such as a history of violence or socio-personal traits that make one vulnerable to discrimination (e.g., mental illness, belonging to certain ethnicities, substance consumption, being foreign). Such factors may increase the possibility of suffering situations of abuse. Protective factors are especially important in counteracting these exclusionary and potentially segregating situations, and in preventing recidivism and the perpetuation of gender-based violence in its different forms.

The prison context must ensure an increase in women’s participation in the various treatment programmes, as well as in socio-educational activities. Such participation not only responds to individual needs but is a key strategy of social sustainability to promote equality and social cohesion. These interventions are associated with the acquisition of knowledge, skills, tools, and strategies necessary not only for inclusive processes, autonomy, and participation but also for empowerment and the ability to identify and face disruptive situations that generate violence and unequal power relationships.

It is also crucial to develop more prison policies that foster equality in the prison environment, consistent with the principles of sustainable human development in this context. Further, means and resources that contribute to these processes and to quality education, re-education, and socio-educational action/intervention are essential. As our study shows, these actions emerge as the core processes of preparation for freedom, which encourage human beings’ social reintegration, emancipation, and freedom.

## 6. Study Limitations

Research in the prison environment is extremely complex, due not only to the limitations of access inherent in a punitive context but also to the multiple administrative requirements and difficulties involved in accessing the sample. Further, the breadth and heterogeneity of the participating population—in age, national origin, and life-course—corresponds to the real prison situation, constraining our analysis to focus on general trends rather than specific comparisons among subgroups. The results should thus be interpreted with caution, without considering women in prison as a homogeneous group.

## Figures and Tables

**Table 1:** Sample distribution by country of birth.

		Frequency	Percentage
Country of birth	Spain	222	71.61%
	Rest of Europe	16	5.16%
	Latin America	58	18.71%
	Other	14	4.52%
	Total	310	100%
Marital status	Married	63	20.45%
	Common law marriage	45	14.61%
	Single	125	40.58%
	Separated/divorced	68	22.08%
	Widowed	7	2.27%
	Total	308	100%
Type of facility	Open regime prison	42	13.55%
	Social Integration Centre (CIS)	160	51.61%
	External Mothers Unit	14	4.52%
	Therapeutic community outside prison	18	5.81%
	Not in a prison	76	24.52%
	Total	310	100%
Sentencing regime	Special second degree (Art. 100.2)	25	8.09%
	Third degree	263	85.11%
	On parole	21	6.80%
	Total	309	100%

Note: The authors.

**Table 2:** Relationship of having partners and children to the prevalence of abuse.

		Abuse			χ^2^	gl	*p*	(Φ)
		No	Yes	Total				
Stable partner	No	38	61	99	5.294	1	0.021	0.149
	Yes	101	91	192				
	Total	139	152	291				
Children	No	22	28	50	0.286	1	0.593	0.076
	Yes	117	126	243				
	Total	139	154	293				

Note: The authors.

**Table 3:** Relationship between employment in the year prior to going to prison and prevalence of abuse.

		Abuse			χ^2^	gl	*p*	(Φ)
		No	Yes	Total				
Employment in the year prior to going to prison	No	59	48	107	4.078	1	0.043	0.147
	Yes	78	104	182				
	Total	137	152	289				

Note: The authors.

**Table 4:** Relationship between substance dependency and prevalence of abuse.

		Abuse			χ^2^	gl	*p*	(Φ)
		No	Yes	Total				
Substance dependency (excl. tobacco, alcohol, and MMP)	No	116	108	224	7.203	1	0.007	0.175
	Yes	23	46	69				
	Total	139	154	293				

Note: The authors.

**Table 5:** Relationship of recidivism and sanctions received while serving the sentence to the prevalence of abuse.

		Abuse			χ^2^	gl	*p*	(Φ)
		No	Yes	Total				
Sanction while serving your sentence	No	109	102	211	6.759	1	0.009	0.018
	Yes	20	41	61				
	Total	129	143	272				
Recidivism profile	No	100	118	218	0.842	1	0.359	0.054
	Yes	39	36	75				
	Total	139	154	293				

Note: The authors.

**Table 6:** Relationship between participating in educational or socio-educational activities and prevalence of abuse.

		Abuse			χ^2^	gl	*p*	(Φ)
		No	Yes	Total				
Received primary or secondary education in a prison environment	No	51	75	126	3.885	1	0.049	0.131
	Yes	85	78	163				
	Total	136	153	289				
Participated in activities on daily life skills	No	79	62	141	8.04	1	0.005	0.166
	Yes	60	92	152				
	Total	139	154	293				
Participation in socio-educational or gender-related programmes	No	83	67	150	7.679	1	0.006	0.162
	Yes	56	87	143				
	Total	139	154	293				

Note: The authors.

## Data Availability

Data supporting the study findings are subject to ethical and legal restrictions due to the sensitive nature of information collected from incarcerated individuals. Therefore, the datasets are not publicly available. Aggregated results are included in the article.
